# Atrial fibrillation ablation using real-time three-dimensional transesophageal echocardiography as the primary intraprocedural imaging modality: a single-center feasibility study

**DOI:** 10.1007/s10840-026-02355-1

**Published:** 2026-05-21

**Authors:** Casey Willaford, Malcolm Woollett, Ian Woollett

**Affiliations:** 1 Sentara Cardiology Specialists, Norfolk, VA USA; 2 University of Virginia, Charlottesville, VA USA; 3 Macon & Joan Brock Virginia Health Sciences at Old Dominion University, Norfolk, VA USA; 4 Sentara Cardiovascular Research Institute, Norfolk, VA USA

**Keywords:** Atrial fibrillation ablation, Transesophageal echocardiography, Three-dimensional echocardiography, Intraprocedural imaging, Pulsed field ablation

## Abstract

**Background:**

Intracardiac echocardiography (ICE) is commonly used during atrial fibrillation (AF) ablation to facilitate transseptal access, catheter positioning, and intraprocedural safety monitoring but adds procedural cost and requires additional vascular access. Real-time three-dimensional transesophageal echocardiography (3D TEE) offers high-quality imaging without disposable intravascular catheters; however, its use as a primary intraprocedural imaging modality during contemporary AF ablation has been limited. This study evaluated the feasibility, safety, and procedural efficiency of using real-time 3D TEE in place of ICE for intraprocedural guidance during AF ablation.

**Methods:**

This single-center feasibility study included 128 consecutive AF ablation procedures performed over an eight-month pilot period using real-time 3D TEE as the primary intraprocedural imaging modality. TEE was used for transseptal puncture guidance, catheter visualization, and intraprocedural safety monitoring. Procedural characteristics, fluoroscopy utilization, acute procedural success, complications, and same-day discharge rates were analyzed.

**Results:**

Acute procedural ablation success was achieved in all cases. Procedures included de novo and repeat ablations, cases requiring lesion sets beyond pulmonary vein isolation, and concomitant left atrial appendage occlusion. No major acute complications, including pericardial effusion requiring intervention, clinically apparent esophageal injury, cerebrovascular events, or in-hospital mortality, were observed. Median total procedure time was 40 min (interquartile range (IQR) 32–50), median fluoroscopy time was 8.6 min (IQR 6.2–11.0), and median radiation dose was 39.1 mGy (IQR 23.2–78.9). Same-day discharge was achieved in 87.5% of procedures.

**Conclusion:**

Real-time 3D TEE can be used as the primary intraprocedural imaging modality for AF ablation, providing effective guidance for transseptal access, catheter positioning, and safety monitoring without reliance on ICE. In centers with advanced echocardiographic expertise and appropriate training frameworks, this approach may represent a viable alternative to ICE with potential advantages in procedural workflow and resource utilization.

**Capsule Summary:**

Real-time three-dimensional transesophageal echocardiography can be used as the primary intraprocedural imaging modality during atrial fibrillation ablation, providing effective guidance for catheter manipulation and safety monitoring while avoiding disposable intracardiac echocardiography catheters and additional vascular access.

**Supplementary Information:**

The online version contains supplementary material available at https://doi.org/10.1007/s10840-026-02355-1.

## Introduction

Intracardiac echocardiography (ICE) is widely used in the United States to facilitate atrial fibrillation (AF) ablation, where it provides real-time imaging for transseptal puncture, catheter positioning, and monitoring for procedural complications while reducing fluoroscopy exposure [1–5]. Over the past decade, ICE has become an integral component of contemporary AF ablation workflows at many centers. However, ICE requires an additional venous access site and a disposable imaging catheter for each procedure, introducing potential vascular risk and substantial per-case cost [3, 6]. These factors have limited the adoption of ICE in many healthcare systems outside the United States and may increasingly influence procedural workflows as AF ablation expands into ambulatory surgical center environments [7–9]. 

Transesophageal echocardiography (TEE), including real-time three-dimensional (3D) imaging, has previously been described for guidance of transseptal puncture and left atrial instrumentation during AF ablation [10, 11]. Early studies established the ability of real-time 3D TEE to visualize transseptal access, pulmonary vein anatomy, and left atrial structures [11], and demonstrated reductions in fluoroscopy use and procedural times with TEE-guided workflows [10]. More recently, TEE has been applied to contemporary pulsed field ablation procedures, with initial reports demonstrating feasibility and the potential for fluoroless procedural approaches [12, 13]. Despite these experiences, systematic evaluation of real-time 3D TEE as the primary intraprocedural imaging modality throughout modern AF ablation workflows—including continuous catheter manipulation, device configuration, and pulmonary vein engagement—remains limited.

Accordingly, we sought to evaluate the feasibility, safety, and procedural efficiency of using real-time 3D TEE in place of ICE for intraprocedural guidance during AF ablation in a consecutive series of AF ablation procedures.

## Methods

### Study design and patient population

This study was a single-center retrospective analysis of consecutive atrial fibrillation ablation procedures performed over an eight-month quality improvement pilot in which real-time three-dimensional transesophageal echocardiography (TEE) was used as the primary intraprocedural imaging modality. All AF ablation procedures during this interval were performed using TEE rather than intracardiac echocardiography (ICE) for intraprocedural imaging guidance. Patients undergoing ablation were assessed according to standard clinical practice for contraindications to transesophageal echocardiography; no patients were excluded on this basis during the study period. This retrospective study was approved by the Eastern Virginia Medical School/Old Dominion University Institutional Review Board (IRB #26-03-WC-0052) with waiver of informed consent and HIPAA authorization. During the study period, two patients were found to have left atrial appendage thrombus on pre-procedural TEE and ablation was deferred; these cases were excluded from the present analysis.

The unit of analysis was the ablation procedure; a small number of patients underwent more than one AF ablation during the study period and are therefore represented more than once in the dataset. Study data were collected and managed using REDCap (Research Electronic Data Capture), a secure, web-based software platform designed to support data capture for research studies. [14, 15]

### Anesthesia and intraprocedural monitoring

All procedures were performed under general anesthesia administered by a certified registered nurse anesthetist operating under anesthesiologist supervision, with endotracheal intubation. Esophageal temperature probes or arterial lines were not routinely used. Glycopyrrolate (0.4 mg) was administered prior to ablation in patients without a pacemaker or implantable cardioverter-defibrillator to mitigate vagally mediated bradycardia associated with pulsed field ablation [16]. 

### Transesophageal echocardiography workflow

Following induction of anesthesia and endotracheal intubation, a TEE probe was inserted by the attending electrophysiologist in the standard fashion. The left atrial appendage was evaluated to exclude thrombus, and a limited baseline echocardiographic assessment was performed. The probe was then handed off to a trained non-physician member of the procedural team who remained positioned at the head of the bed while the electrophysiologist prepared and proceeded with vascular access and ablation. In this workflow, intraprocedural probe manipulation was performed by a non-physician team member with deliberate and supervised training in diagnostic echocardiography, functioning under continuous physician direction; clinical responsibility for image interpretation and all procedural decisions remained with the attending electrophysiologist throughout.

Throughout the procedure, TEE image acquisition and probe manipulation were performed under continuous, direct supervision of the electrophysiologist. Two-dimensional and predominantly three-dimensional TEE imaging was used to obtain operator-directed views for transseptal puncture, catheter positioning, catheter configuration, and monitoring for procedural complications. TEE images were displayed on the primary procedural monitor to facilitate continuous integration with fluoroscopic and electroanatomic mapping data. All imaging was done using a Phillips EPIQ CVX echo machine and a X8-2t TEE probe.

### Vascular access and transseptal puncture

In most cases, a single right femoral venous sheath was placed. When activation mapping was required for atrial flutter or organized atrial tachycardia, a second venous sheath was placed in the left femoral vein and a coronary sinus catheter was used for timing reference.

Transseptal puncture was performed under TEE guidance using bicaval, short-axis, and real-time 3D imaging views. After left atrial access was obtained, the transseptal sheath was exchanged for the appropriate delivery sheath based on the ablation system used.

### Ablation strategy

The primary ablation strategy was pulmonary vein isolation (PVI) for de novo AF ablation procedures and re-isolation of reconnected pulmonary veins for repeat procedures. Additional left atrial lesion sets beyond PVI (PVI+) were performed at the operator’s discretion based on clinical and anatomic considerations, including atrial size, AF chronicity, substrate characteristics, and inducible arrhythmias. Cavotricuspid isthmus (CTI) ablation for typical atrial flutter was performed when clinically indicated and was analyzed separately from PVI+ lesion sets.

The majority of procedures were performed using a pentaspline pulsed field ablation system (Boston Scientific, Farapulse). A small minority of procedures were performed using a focal pulsed field ablation system (Medtronic, Affera). Electroanatomic mapping was performed using the NavX system (Abbott) in all cases except those using the Affera platform.

**Fig. 1:**
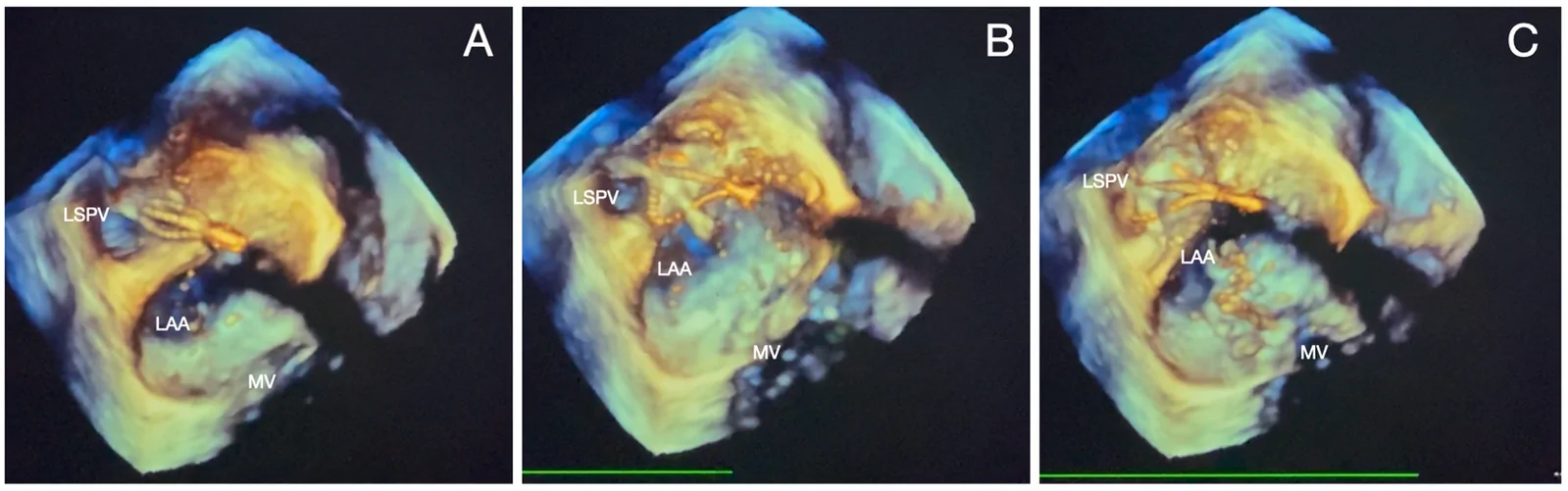
Real-time three-dimensional transesophageal echocardiography demonstrating a three-panel progression of a pentaspline pulsed field ablation catheter during left atrial navigation. **A** shows the catheter in the undeployed configuration within the left atrium. **B** demonstrates expansion into the basket configuration. **C** shows advancement and engagement of the deployed basket into the ostium of the left superior pulmonary vein (LSPV). The left atrial appendage opening (LAA), left atrial appendage ridge, and mitral valve (MV) are also visible, providing anatomic context for catheter orientation

**Fig. 2:**
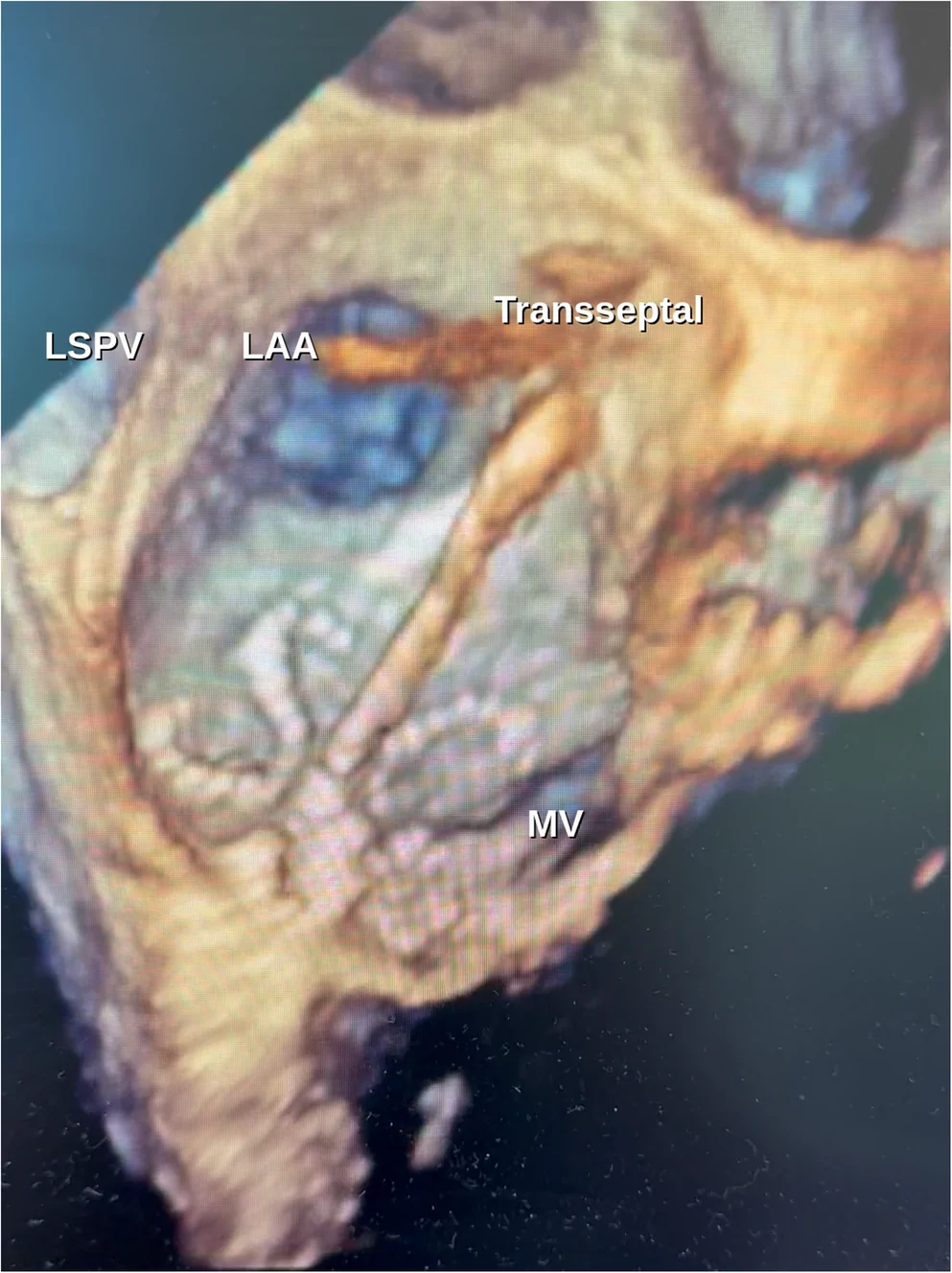
Real-time three-dimensional transesophageal echocardiography image showing the pentaspline pulsed field ablation catheter deployed in the flower configuration on the mitral valve annulus while performing an anterior mitral valve flutter ablation. The left atrial appendage (LAA), mitral valve (MV), left superior pulmonary vein (LSPV) and transseptal puncture site are also visible, illustrating their anatomic relationship

**Fig. 3:**
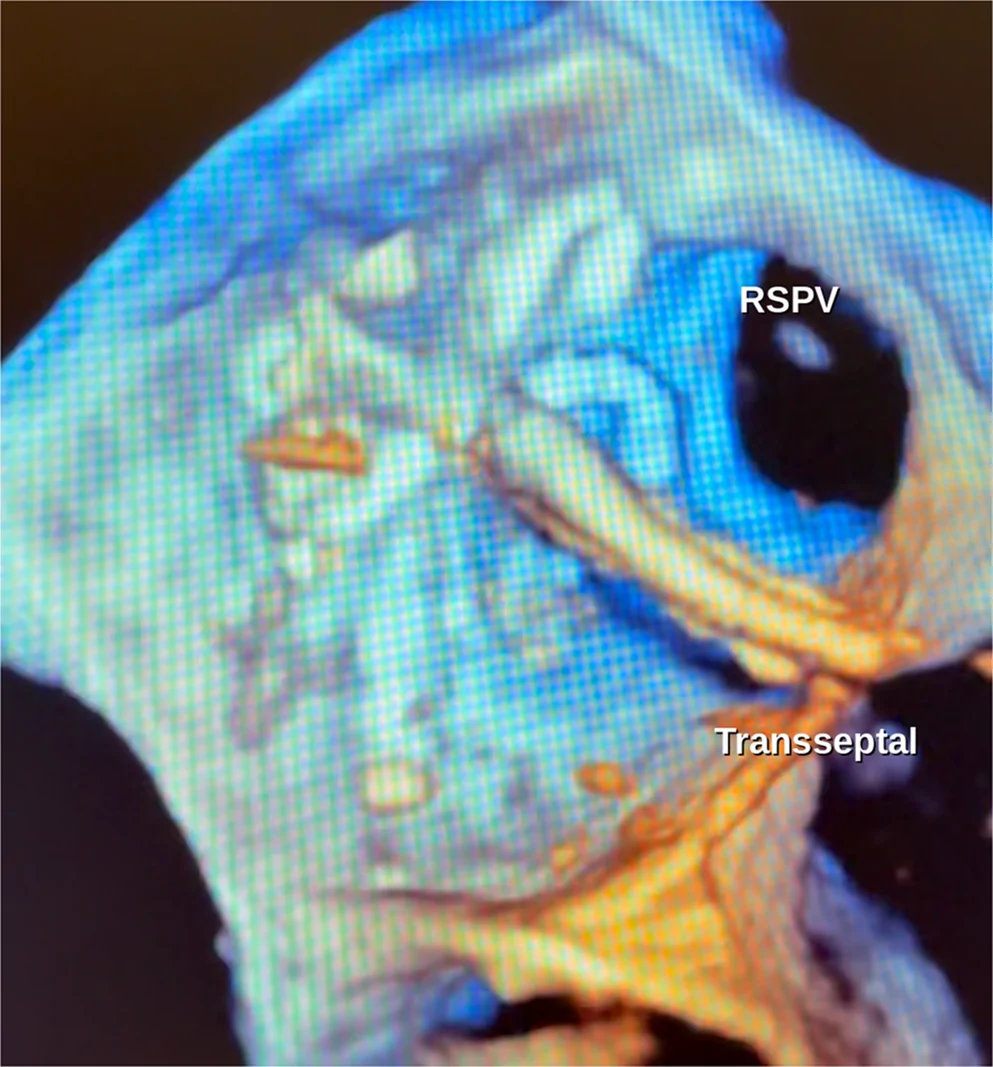
Real-time three-dimensional transesophageal echocardiographic visualization of the pentaspline pulsed field ablation catheter in the flower configuration positioned along the posterior wall of the left atrium, highlighting catheter–wall apposition during posterior wall ablation and showing the right superior pulmonary vein (RSPV) and transseptal location

During ablation, real-time 3D TEE was used to follow the ablation catheter throughout the left atrium, assess catheter contact, guide catheter rotation and configuration, and minimize fluoroscopy use. When CTI ablation was performed, catheter position was similarly visualized using both two-dimensional and three-dimensional TEE imaging. Representative examples of pentaspline catheter configuration changes and pulmonary vein engagement are shown in Figs. 1, 2 and 3 and Supplementary Videos 1, 2, 3, 4, 5, 6, 7 and 8.

### Safety monitoring and procedural completion

Periodic TEE assessments were performed during the procedure to evaluate for pericardial effusion, with additional imaging performed if hypotension or hemodynamic instability occurred. At the conclusion of the procedure, absence of a pericardial effusion was confirmed prior to removal of the TEE probe. Protamine was administered, and the ablation sheath was removed in the electrophysiology laboratory using a figure-of-eight suture for venous hemostasis. All patients underwent standardized follow-up at approximately two weeks post-procedure, including nurse assessment with 12-lead electrocardiogram, evaluation for vascular access complications, and systematic screening for symptoms suggestive of esophageal injury, including dysphagia, odynophagia, or persistent throat discomfort.

### Outcomes and definitions

Acute procedural success was defined as achievement of the intended ablation endpoints, including electrical isolation of all targeted pulmonary veins and completion of additional planned lesion sets when performed. Procedural complications were assessed acutely and included pericardial effusion requiring intervention, clinically apparent esophageal injury, cerebrovascular events, or in-hospital mortality.

Procedural efficiency metrics included physician TEE time (defined as time the physician spent passing the TEE probe and obtaining baseline images before passing off the probe and moving to get femoral venous access), ablation time (defined as initial femoral venous access to final sheath removal), total procedure time (TEE + ablation), fluoroscopy time, and radiation dose. Same-day discharge was recorded as a binary outcome.

## Results

### Study population and procedural characteristics

A total of 128 consecutive atrial fibrillation ablation procedures were performed using a real-time 3D TEE–guided workflow (130 cases were originally scheduled, but 2 cases were deferred due to detection of LAA thrombus on the initial TEE). The mean patient age was 68.8 ± 9.8 years, and 41.4% of procedures were performed in female patients. 25% of cases occurred in patients who had undergone one or more AF ablation procedures in the past (redo cases). Concomitant left atrial appendage occlusion (LAAO) was performed in 14.8% of procedures.

Pulmonary vein isolation alone was performed in 50.0% of procedures, while 50.0% included additional left atrial lesion sets beyond PVI (PVI+). Cavotricuspid isthmus ablation was performed in 14.8% of cases. In the majority of procedures (80.5%), ablation was performed using a single femoral venous sheath (Table 1).

**Table 1:** Baseline Patient and Procedural Characteristics

Characteristic	Value
Number of procedures	128
Age, years (mean ± SD)	68.8 ± 9.8
Female sex, n (%)	53 (41.4%)
Redo ablation, n (%)	32 (25.0%)
Concomitant LAAO, n (%)	19 (14.8%)
PVI only, n (%)	64 (50.0%)
PVI+, n (%)	64 (50.0%)
CTI ablation, n (%)	19 (14.8%)
Single femoral venous sheath, n (%)	103 (80.5%)
Ablation system: Farapulse, n (%)	126 (98.4%)
Ablation system: Affera, n (%)	2 (1.6%)

Baseline demographic and procedural characteristics for 128 consecutive atrial fibrillation ablation procedures performed using real-time three-dimensional transesophageal echocardiography as the primary intraprocedural imaging modality

### Procedural success and safety

Acute procedural success was achieved in all cases as defined by achievement of intended pulmonary vein isolation and completion of planned ablation lesion sets without intraprocedural conversion of imaging strategy. For concomitant cases, LAAO device implant success rates were analyzed separately with successful device implantation in 18/19 cases (95%); in one case LAAO device implantation was deferred due to anatomic unsuitability of the appendage although the AF ablation was successfully completed. No major acute procedural complications were observed, including pericardial effusion requiring intervention, clinically apparent esophageal injury or pain, vascular injury requiring surgical repair, cerebrovascular events, or in-hospital mortality (Table 2).

**Table 2:** Acute procedural outcomes

Outcome	*N* (%)
Acute procedural success	
- Ablation success	128/128 (100%)
- LAAO success	18/19 (95%)
Major Acute Complications	
- Pericardial effusion requiring intervention	0 (0%)
- Clinically apparent esophageal injury	0 (0%)
- Vascular injury requiring surgical repair	0 (0%)
Stroke/TIA	0 (0%)
In-hospital mortality	0 (0%)

Acute procedural success was defined as achievement of intended pulmonary vein isolation and completion of planned lesion sets or concomitant procedures without intraprocedural conversion of imaging strategy. Major acute complications included pericardial effusion requiring intervention, clinically apparent esophageal injury or persistent esophageal symptoms, vascular injury requiring surgical repair, cerebrovascular events, or in-hospital death

### Procedural efficiency and fluoroscopy utilization

Median physician TEE time was 5 min (IQR 4–6). Median ablation procedure time was 35 min (IQR 28–44), and the median total procedure time (TEE + ablation) was 40 min (IQR 32–50). These times reflect a high-volume pulsed field ablation practice and may not be representative of all atrial fibrillation ablation workflows. Median fluoroscopy time was 8.6 min (IQR 6.2–11.0), with a median radiation dose of 39.1 mGy (IQR 23.2–78.9) (Table 3). Dynamic catheter manipulation, configuration changes, and rotational alignment were best appreciated on real-time three-dimensional imaging and are illustrated in Figs. 1, 2 and 3, and Supplementary Videos 1, 2, 3, 4, 5, 6, 7 and 8.

**Table 3:** Procedural efficiency and fluoroscopy metrics

Metric	Median (IQR)
Physician TEE time, minutes	5 (4–6)
AF ablation time, minutes	35 (28–44)
Total procedure time, minutes (TEE + ablation)	40 (32–50)
Fluoroscopy time, minutes	8.6 (6.2–11.0)
Radiation dose, mGy	39.1 (23.2–78.9)
Same-day discharge, n (%)	112 (87.5%)

Procedural efficiency, fluoroscopy utilization, and discharge outcomes for atrial fibrillation ablation performed with real-time three-dimensional transesophageal echocardiographic guidance. Values are presented as median (interquartile range) unless otherwise specified

### Impact of concomitant left atrial appendage occlusion

Procedures that included concomitant left atrial appendage occlusion were associated with longer median procedure times compared with AF ablation alone. Fluoroscopy time and radiation dose were also higher in cases with concomitant LAAO. Same-day discharge was achieved in the majority of cases in both groups (Table 4).

**Table 4:** Impact of concomitant left atrial appendage occlusion

Metric	AF Ablation Alone (*n* = 109)	AF Ablation + LAAO (*n* = 19)
Physician TEE time, minutes	5 (4–6)	5 (4–6)
Procedure time, minutes	32 (28–41)	50 (43–56)
Total procedure time, minutes	37 (33–46)	55 (49–60)
Fluoroscopy time, minutes	8.1 (6.0–10.5)	11.2 (8.9–14.3)
Radiation dose, mGy	35.4 (22.1–69.8)	78.9 (52.4–121.6)
Same-day discharge, n (%)	98 (89.9%)	14 (73.7%)

Procedural efficiency, fluoroscopy utilization, radiation exposure, and discharge outcomes stratified by the presence or absence of concomitant LAAO. Subgroup data stratified by concomitant left atrial appendage occlusion are presented descriptively to illustrate workflow adaptability and procedural impact; no formal statistical comparison between groups was performed. Values are presented as median (interquartile range) unless otherwise specified

### Discharge outcomes

Overall, 87.5% of procedures were performed with same-day discharge.

## Discussion

### Principal findings

In this single-center feasibility study of 128 consecutive atrial fibrillation ablation procedures, real-time three-dimensional transesophageal echocardiography was successfully implemented as the primary intraprocedural imaging modality in place of intracardiac echocardiography. The workflow was associated with a 100% acute procedural success rate for ablation goals, absence of major acute complications and procedural efficiency metrics that fall within ranges reported in contemporary AF ablation practice. These findings were observed across a heterogeneous cohort that included de novo and repeat ablations, procedures requiring lesion sets beyond pulmonary vein isolation, and cases performed concomitantly with left atrial appendage occlusion. This study was designed as a feasibility evaluation and was not intended to compare intraprocedural imaging modalities or to assess superiority; rather, the objective was to determine whether a real-time three-dimensional TEE–guided workflow could be implemented across a heterogeneous spectrum of contemporary AF ablation procedures without evident compromise in procedural efficiency or acute safety.

Importantly, this experience demonstrates that real-time 3D TEE can be used not merely as an adjunct for transseptal access or an anatomic reference, but as an integrated imaging modality capable of guiding catheter manipulation, device configuration, and procedural safety monitoring throughout the entirety of a modern AF ablation procedure. The high rate of same-day discharge further supports the feasibility of incorporating this approach into a high-throughput clinical practice.

### Relationship to prior literature and novelty of the approach

Transesophageal echocardiography has long played a role in AF ablation, particularly for exclusion of left atrial appendage thrombus and guidance of transseptal puncture. Early reports describing real-time three-dimensional TEE demonstrated its ability to provide detailed visualization of left atrial anatomy and septal structures, and select studies have suggested that TEE-guided workflows may reduce fluoroscopy exposure during AF ablation. However, much of the existing literature consists of early feasibility studies, small case series, or workflows in which TEE was used intermittently or for limited procedural steps rather than as a continuous intraprocedural imaging modality.

More recently, three-dimensional and four-dimensional intracardiac echocardiography systems have been introduced and are increasingly used during atrial fibrillation ablation, offering real-time volumetric imaging capabilities that address some of the traditional imaging limitations of conventional ICE [17, 18]. While these platforms can provide high-quality intraprocedural visualization, they rely on disposable imaging catheters and are associated with substantially higher per-procedure costs compared with standard ICE, which itself already represents a significant per-procedure disposable cost of several thousand dollars per case. In contrast, real-time three-dimensional transesophageal echocardiography provides volumetric imaging without the need for disposable intravascular imaging catheters.

The procedural times, fluoroscopy utilization, and acute outcomes observed in this series are consistent with those reported in contemporary real-world PFA registries [19, 20] and fall within ranges observed in a recent randomized trial comparing ICE and TEE for preprocedural thrombus screening during AF ablation [21], noting that the latter study addressed a distinct clinical question. No direct comparison was performed in the present study, and these observations should be interpreted as contextual rather than comparative. Accordingly, substitution of ICE with real-time 3D TEE did not appear to adversely impact procedural workflow or acute procedural endpoints, although this study was designed as a feasibility evaluation and was not intended to provide a direct comparative assessment against ICE-guided workflows.

The application of TEE to pulsed field ablation represents a particularly timely evolution of this imaging approach. While the majority of published TEE-guided ablation experience predates widespread PFA adoption, recent reports have begun to evaluate TEE specifically in the context of pulsed field energy delivery. Liu et al. recently described their initial experience with TEE-guided PFA, demonstrating procedural feasibility and safety [12], while Zhou et al. reported a fluoroless workflow for PFA procedures guided entirely by TEE without fluoroscopic imaging [13]. These contemporary reports, combined with earlier work demonstrating the utility of TEE for transseptal puncture guidance [10] and detailed pulmonary vein anatomic visualization [11], support the broader application of real-time 3D TEE as a viable primary imaging modality for contemporary AF ablation workflows. The present series extends these observations by demonstrating sustained procedural efficiency and safety across a heterogeneous case mix including repeat ablations, complex lesion sets, and concomitant procedures, suggesting that the TEE-guided approach is adaptable to diverse clinical scenarios rather than limited to selected cases.

### Workflow and procedural implications

The use of real-time 3D TEE had several practical implications for procedural workflow. Continuous echocardiographic visualization allowed real-time assessment of catheter position, orientation, and configuration within the left atrium, reducing reliance on fluoroscopy for catheter advancement, rotation, and deployment. Although this study was not designed to formally assess a learning curve, fluoroscopy utilization appeared to decrease over the course of the pilot period, and in later cases fluoroscopy was often limited to only a few minutes, consistent with increasing operator familiarity and confidence with real-time three-dimensional TEE–guided catheter manipulation. This was particularly relevant during pentaspline pulsed field ablation, where assessment of basket and flower configurations and rotational alignment at the pulmonary vein ostia can be challenging using fluoroscopy alone. These configuration changes and anatomic relationships are illustrated in Figs. 1, 2 and 3, with dynamic examples provided in the Supplementary Videos.

In practice, two-dimensional imaging served as the primary modality for baseline assessment and transseptal puncture, with three-dimensional imaging used predominantly for intraprocedural catheter navigation; the need to revert to two-dimensional imaging for recentering decreased progressively as team familiarity with real-time three-dimensional visualization developed. Importantly, the fluoroscopy metrics reported here reflect an initial adoption period and are likely not representative of the workflow’s ultimate efficiency ceiling. In the six months following completion of this analysis, fluoroscopy use has continued to decline with accumulating team experience, with many procedures now completed with minimal fluoroscopic guidance — an observation that suggests the reported metrics may meaningfully understate the fluoroscopy reduction achievable with an established 3D TEE-guided workflow.

Beyond catheter manipulation, 3D TEE provided valuable anatomic context through simultaneous visualization of the interatrial septum, left atrial appendage, mitral valve apparatus, and pulmonary vein ostia. This spatial orientation facilitated navigation to both left- and right-sided pulmonary veins and was especially helpful in cases requiring more complex lesion sets or concomitant left atrial appendage occlusion. Despite increased procedural complexity in these cases, procedural efficiency was maintained.

From a vascular access perspective, use of TEE in place of ICE eliminated the need for routine placement of a dedicated venous sheath for intracardiac imaging in the majority of cases. Most procedures were performed with a single femoral venous sheath, simplifying access and potentially influencing vascular access-related complications without compromising intraprocedural imaging capability.

### Safety considerations and imaging tradeoffs

Use of transesophageal echocardiography as the primary intraprocedural imaging modality introduces a distinct safety profile compared with intracardiac echocardiography. TEE carries a small but recognized risk of esophageal injury or perforation and theoretical concerns regarding transducer heating during prolonged three-dimensional imaging acquisition [22–24]. In this series, no clinically apparent esophageal injury, esophageal pain, or probe-related complications were observed. While systematic post-procedural esophageal evaluation was not performed, the absence of clinically evident esophageal sequelae supports the short-term safety of this approach in a carefully selected population.

ICE, in contrast, requires additional venous access and intravascular catheter manipulation, introducing a different set of potential risks, including hematoma, pseudoaneurysm formation, and the need for vascular closure devices [25, 26]. By eliminating routine placement of a dedicated venous sheath for intracardiac imaging, the TEE-guided workflow reduced the number of vascular access sites required for most procedures. Although this study was not designed to compare complication rates between imaging strategies, these differing risk profiles highlight the importance of considering imaging tradeoffs rather than assuming a uniform safety advantage of one modality over another.

Real-time 3D TEE also enabled continuous surveillance for pericardial effusion throughout the procedure, with targeted reassessment in response to hemodynamic changes and routine confirmation of the absence of effusion at case completion. This safety monitoring capability is comparable to that provided by ICE and reinforces the feasibility of TEE as an alternative intraprocedural imaging modality.

A threshold safety and workflow consideration when evaluating transesophageal echocardiography as a primary intraprocedural imaging modality is whether its use introduces an additional invasive procedural step. Practice patterns for pre-ablation thrombus exclusion vary across centers, with some routinely performing TEE, others using cardiac CT or ICE-based strategies, and some omitting imaging in selected anticoagulated patients [1, 26]. In centers where a screening TEE is already performed prior to transseptal access, extending TEE from thrombus exclusion to continuous intraprocedural guidance does not require an additional probe insertion; however, it may increase probe dwell time compared with screening-only workflows [22]. Because the prevalence of left atrial or left atrial appendage thrombus remains low but non-zero despite therapeutic anticoagulation [1, 27], a TEE-guided workflow allows appendage assessment to occur within the same intraprocedural imaging session when such screening is performed. The present study was not designed to evaluate thromboembolic outcomes, and no conclusions regarding comparative stroke prevention strategies can be drawn from this analysis.

### Resource utilization and cost considerations

Beyond procedural feasibility, the choice of intraprocedural imaging modality has important implications for procedural resource utilization. Intracardiac echocardiography requires a single-use disposable imaging catheter for each case. Reported pricing for disposable ICE catheters typically range from approximately $2,000 to $3,500 per procedure, based on publicly available device pricing and institutional contracting estimates, though costs vary by catheter platform and center. In high-volume electrophysiology practices, these per-case expenditures represent a recurring disposable cost that aggregates across operators and annual procedural volumes.

In contrast, transesophageal echocardiography relies on reusable imaging equipment that is widely available in electrophysiology laboratories and operating rooms. Although probe reprocessing incurs nominal operational expense, substitution of a reusable TEE platform for a disposable intravascular imaging catheter has clear implications for procedural supply utilization. The present study was not designed to perform a formal cost-effectiveness analysis; however, elimination of routine ICE catheter use represents a meaningful shift in per-case disposable resource allocation.

In addition to disposable supply considerations, use of TEE in place of ICE may influence vascular access complexity. ICE-guided workflows typically require placement of a dedicated venous sheath for intracardiac imaging. In this series, the majority of procedures were performed using a single femoral venous sheath. Although access-site complication rates were not formally compared, reduction in the number of venous access sites may simplify procedural workflow and influence access-related resource utilization.

The choice of imaging modality may also affect procedural staffing logistics in centers where real-time TEE guidance during concomitant left atrial appendage occlusion requires dedicated imaging physician coverage. In the workflow described here, intraprocedural TEE imaging was performed within a structured, physician-directed team framework without requiring a separate imaging physician operator. TEE imaging was conducted by a non-physician team member whose responsibilities extended beyond procedural imaging and included other clinical and operational duties, and therefore did not represent an additional dedicated staffing resource. While personnel models vary across institutions and formal economic modeling was beyond the scope of this analysis, this approach may offer scheduling flexibility and influence incremental procedural staffing requirements in certain practice environments.

As atrial fibrillation ablation continues to expand globally and increasingly into ambulatory procedural settings [9], imaging strategies that maintain procedural safety and efficiency while altering disposable resource requirements may have growing relevance. Further comparative and economic studies are warranted to better define the broader system-level implications of these approaches.

### Generalizability and implementation considerations

A critical consideration for broader adoption of this workflow is the level of expertise required to consistently obtain high-quality real-time three-dimensional TEE images of the pulmonary veins. Acquisition of these views requires advanced probe manipulation skills and three-dimensional spatial understanding that extends beyond routine diagnostic TEE imaging. In the present study, intraprocedural imaging was performed by a team member with substantial prior experience in comprehensive diagnostic TEE, which likely contributed to reliable catheter visualization across a wide range of patient anatomies.

It is important to emphasize that proficiency in transesophageal echocardiography reflects substantial physician training and experience, particularly among cardiologists and cardiac anesthesiologists. In the workflow presented, intraprocedural TEE imaging was performed under the continuous direction and supervision of the attending physician, with clinical responsibility for image interpretation and procedural decision-making remaining entirely physician-led. Any extension of probe manipulation responsibilities to non-physician team members should occur within a structured, physician-directed framework and should not be interpreted as advocating for independent performance or interpretation of TEE. In this workflow, intraprocedural probe manipulation functioned analogously to camera assistance during minimally invasive surgery, with continuous physician direction and real-time interpretive oversight, rather than independent image acquisition or diagnostic interpretation.

Although intraprocedural 3D TEE imaging in this series was performed by a single non-physician team member, similar workflows have been adopted at other centers using appropriately trained personnel from a variety of clinical backgrounds [28]. The personnel structure used in the present study reflects our institutional practice model. Successful implementation requires a dedicated and capable individual, a well-defined training pathway, and a structured competency assessment framework. At our institution, a formal training plan and competency checklist were developed to support this workflow and could be adapted by other centers with established expertise in advanced transesophageal echocardiography.

Importantly, this approach does not rely on additional physician operators dedicated solely to imaging, which would be resource-intensive and impractical in many settings. Rather, feasibility depends on institutional capability, deliberate training, and close integration between the imager and the proceduralist within existing team structures.

Additionally, although not formally evaluated in the present study, real-time three-dimensional transesophageal echocardiography has since been applied at our institution to guide a variety of other electrophysiology procedures, including ventricular tachycardia, premature ventricular complex, atrial flutter, and Wolff–Parkinson–White ablations. These experiences suggest broader potential applicability of this imaging modality beyond atrial fibrillation ablation, though systematic evaluation in these settings is warranted.

## Limitations

This study has several limitations. This analysis represents the experience of a single operator at a single center and was conducted as a feasibility study without a contemporaneous ICE-guided comparator group. Accordingly, direct comparisons of procedural efficiency, radiation exposure, or complication rates between imaging strategies cannot be made. While patients undergoing atrial fibrillation ablation at our center undergo routine clinical follow-up for arrhythmia recurrence and delayed complications, this particular analysis was limited to acute procedural outcomes, and the study was not designed to evaluate long-term arrhythmia outcomes or delayed complications. Institutional policies and credentialing requirements regarding intraprocedural transesophageal echocardiography, including probe manipulation by non-physician personnel, vary across practice settings and may influence the generalizability of this workflow. Procedural efficiency metrics observed in this series likely reflect operator and team experience with this imaging strategy and may not be immediately generalizable during early adoption phases. Finally, due to the retrospective nature of this study, left atrial dwell time (an increasingly used metric) was not able to be reliably collected but should be considered as a metric in any prospective study going forward.

Finally, the workflow described reflects the experience of a high-volume operator with more than two decades of AF ablation experience working in close collaboration with a non-physician team member with exceptional echocardiographic skills developed over an extended supervised training period. These individual factors likely contributed to the procedural efficiency and safety outcomes observed and may not be readily replicable in centers without comparable operator experience or access to similarly trained imaging personnel. Multicenter evaluation will be essential to determine whether this workflow is reproducible across a broader range of operators, institutions, and team configurations.”

## Conclusions

Real-time three-dimensional transesophageal echocardiography can function as the primary intraprocedural imaging modality for atrial fibrillation ablation, providing effective guidance for transseptal access, catheter positioning, and safety monitoring without reliance on intracardiac echocardiography. In this single-center feasibility study, this approach was associated with high acute procedural success, absence of major acute complications, and efficient procedural performance across a range of ablation strategies, including procedures requiring lesion sets beyond pulmonary vein isolation and concomitant left atrial appendage occlusion.

In centers with advanced transesophageal echocardiographic expertise and appropriate training frameworks, real-time 3D TEE represents a viable alternative to ICE for atrial fibrillation ablation. Broader adoption of this approach may reduce per-procedure disposable costs and expand access to contemporary AF ablation techniques, both in resource-constrained healthcare systems and as atrial fibrillation ablation increasingly transitions to ambulatory surgical center settings [9]. Future multicenter and comparative studies are warranted.

## Electronic Supplementary Material

Below is the link to the electronic supplementary material.

Supplementary Material 1 - Sphere 9 on on MV annulus below LSPV

Supplementary Material 2 - Farapulse on MV annulus below LSPV with LAA behind

Supplementary Material 3 - Farapulse moving from LSPV to LIPV

Supplementary Material 4 - Farapulse on posterior wall

Supplementary Material 5 - Farapulse RSPV to RIPV

Supplementary Material 6 - Farapulse basket engaging LSPV

Supplementary Material 7 - Transseptal puncture

Supplementary Material 8 - Transseptal Pullback

## Data Availability

The datasets generated and analyzed during the current study are not publicly available due to the inclusion of protected patient health information and institutional privacy regulations. Study data were collected and managed using the REDCap (Research Electronic Data Capture) platform and are stored on secure institutional servers. De-identified data may be made available from the corresponding author upon reasonable request and with appropriate institutional review board approval.
